# Standardized quality metric system for structural brain magnetic resonance images in multi-center neuroimaging study

**DOI:** 10.1186/s12880-018-0266-4

**Published:** 2018-09-17

**Authors:** Michael E. Osadebey, Marius Pedersen, Douglas L. Arnold, Katrina E. Wendel-Mitoraj, for the Alzheimer’s Disease Neuroimaging Initiative

**Affiliations:** 1 NeuroRx Research Inc, Montreal, 3575 Parc Avenue, Suite # 5322, Montreal, Quebec, H2X 3P9 Canada; 2 Department of Computer Science, Norwegian University of Science and Technology, Teknologivegen 22, Gjøvik, N-2815 Norway; 3 Montreal Neurological Institute and Hospital, McGill University, 3801 University St, Montreal, Quebec, H3A 2B4 Canada; 4 BrainCare Oy, Finn-Medi 1 PL 2000, Tampere, 33521 Finland; 5 The Alzheimer’s Disease Neuroimaging Initiative, Center for Imaging of Neurodegenerative Disease, San Francisco VA Medical Center, University of California, San Francisco, USA

**Keywords:** Magnetic resonance imaging (MRI), Brain MRI, Image quality, Image moment, Grayscale feature image, Local contrast feature image

## Abstract

**Background:**

Multi-site neuroimaging offer several benefits and poses tough challenges in the drug development process. Although MRI protocol and clinical guidelines developed to address these challenges recommend the use of good quality images, reliable assessment of image quality is hampered by the several shortcomings of existing techniques.

**Methods:**

Given a test image two feature images are extracted. They are grayscale and contrast feature images. Four binary images are generated by setting four different global thresholds on the feature images. Image quality is predicted by measuring the structural similarity between appropriate pairs of binary images. The lower and upper limits of the quality index are 0 and 1. Quality prediction is based on four quality attributes; luminance contrast, texture, texture contrast and lightness.

**Results:**

Performance evaluation on test data from three multi-site clinical trials show good objective quality evaluation across MRI sequences, levels of distortion and quality attributes. Correlation with subjective evaluation by human observers is ≥ 0.6.

**Conclusion:**

The results are promising for the evaluation of MRI protocols, specifically the standardization of quality index, designed to overcome the challenges encountered in multi-site clinical trials.

## Background

Brain imaging studies using magnetic resonance imaging (MRI) system is one of the strongest biomarker candidates for neurological diseases [1–8]. MRI system is highly flexible. A single MRI system examination can be configured to generate several image sequences which can potentially provide high contrast structural information and the connectivity between brain structures [9–11]. The first listing of MRI as criteria for the diagnosis of multiple sclerosis and Alzheimer’s diseases was in 2001 and 2010, respectively [12, 13]. Over the years, advances in technology, strong collaboration among researchers coupled with availability of clinical data encouraged modifications [14–17] to the initial diagnostic criteria. Despite several modifications to the initial diagnostic criteria, MRI criteria maintains a strong position to demonstrate the dissemination of multiple sclerosis disease in space and time and the exclusion of other disorders that can mimic its clinical and laboratory profile [18].

Pharmaceutical companies incorporate multi-center neuroimaging in the design of clinical trials for the treatment and the monitoring of neurological diseases. Multi-site clinical trials has several advantages. Main benefit is the ability to obtain more neuroimaging data per unit time across a wide variety of the patient population [19]. Other benefits include monitoring the progression of disease across a geographically, culturally and environmentally diverse population [20].

Several challenges encountered in multi-center neuroimaging studies result from the differences in MRI system parameters and image reconstruction routines. Two challenges are the variation of scanner technologies across the clinical trials sites and the different magnetic field inhomogeneities produced by a specific MRI model from the same manufacturer [21, 22]. These challenges renders acquired data including image quality evaluation unreliable with high risk of inaccurate diagnosis [20].

There have been proposals to mitigate the effects of these challenges. They include careful coordination, the use of standardized phantom studies and strict quality assurance of recommended imaging protocols across the clinical trial sites [18, 23, 24]. Another mitigation measure is to ensure that end points derived from brain MRI images are subjective and heavily dependent on radiologist interpretation [25]. This approach is grossly inefficient in large scale clinical trials where large volume of data are processed. Conflicting results can result from the variability and lack of reproducibility in the interpretation of data by radiologists [2, 26, 27].

Acquisition and reconstruction of MRI image from k-space data is time consuming relative to other imaging modalities such as X-ray, ultrasound and computed tomography. The consequences which include patient discomfort and motion-related artifacts significantly limits the potential to acquire high quality images [28, 29]. At the post-acquisition stage such as multi-center clinical trials, the challenge is centered on standardizing MRI data from different MRI scanners and different MRI sequences. Quality evaluation beyond the acquisition stage is necessary to evaluate the performance of MRI protocols developed to mitigate the challenges in multi-site neuroimaging.

Most proposed quality evaluation methods focus on the acquisition stage. There are few contributions on post-acquisition quality evaluation of brain MRI images. They include [30] which apply analysis of variance (ANOVA) algorithm to assess the variation of several quality measures with different levels of distortions. The authors in [31] combine the detection of artifacts and estimation of noise level to measure image quality. In [32] null space analysis and just noticeable difference scanning method was proposed as a better quality metric compared to root-mean-square error (RMSE). The popular signal-to-noise ratio (SNR) is the quality metric adopted in [33]. Recently the report in [34] propose a new method which predict brain MRI quality based on five quality attributes. The attributes are lightness, contrast, sharpness, texture details and noise. This report provide brief review of image quality evaluation. Detailed review of generic approaches to image quality evaluation can be found in [35, 36]. Review focussed on medical images is available in [31, 37, 38].

We share the same opinion in [39, 40] which suggest that the design of application-specific, no-reference quality evaluation system is more realistic and practicable than the design of generic image quality evaluation systems. It will be futile to design a generic image quality metric because images possess unique characteristics that distinguish them within and across different classes. Furthermore current state-of-the-art generic quality evaluation algorithms will require significant modifications before application to medical images [37].

Shortcomings of current state-of-the-art quality evaluation algorithm justify the need for new approach to quality evaluation in multi-center clinical trials. The contribution by [33] which adopt SNR is a full reference method based on the assumption that there exists a perfect image. In the real world a perfect image does not exist [37]. There are many definitions of SNR with no clearly defined range of quality index. These characteristics makes it difficult to compare quality measures from different imaging system, modalities and researchers [41]. Quality indices derived from SNR does not always correlate with the performance of observers using the imaging system on the task for which they are intended [41]. The use of SNR for quality evaluation can be said to be diagnostically misleading [42] because it cannot discriminate the quality of two images that are perceptually dissimilar [32]. The adoption of only artifacts and noise in [31] are too few attributes to evaluate the quality of an image. There are two setbacks for ANOVA based technique proposed in [30]. They are the risk of ambiguity in quality measures and the inability to transform the different levels of distortion into a quality index [43].

Image quality evaluation is difficult and complicated. Perceived image quality is influenced by several types of quality attributes and the different attributes influence each other [44]. The proposed method follow the three-step framework reported by Bartleson in 1982 [45]. The first step identify the most significant quality attributes of brain MRI images. They are luminance contrast, texture, texture contrast and lightness. The second step adopt image moments as global threshold to binarize and capture structural information contained in grayscale and contrast feature images derived from brain MRI images. Image moments have been successfully applied in many areas of image analysis such as image denoising [46], speech recognition [47], image normalization [48, 49], reconstruction [50], feature selection [51, 52], content-based image retrieval systems [53] and segmentation [54], [55]. In the third step image quality is evaluated based on structural similarity between the pair of images derived by using different moments to binarize each feature image.

This paper is organized as follows. The next section is the methods section. It include the setup of the experiment, problem formulation and implementation of the algorithm. The results of performance evaluation are presented in “Results” section and discussed in “Discussion” section. “Discussion” section highlight the limitations of our proposed method and the future work to overcome these limitations. “Conclusions” section concludes this report.

## Methods

### Sources of data

Data used for the performance evaluation of the proposed method were obtained from NeuroRx research Inc. (https://www.neurorx.com), BrainCare Oy. (http://braincare.fi/) and the Alzheimer’s disease neuroimaging initiative (ADNI) database (www.adni.loni.usc.edu).

NeuroRx research Inc. is an international clinical research organization dedicated to working with the pharmaceutical industry to facilitate clinical trials of new drugs for multiple sclerosis (MS) and other neurological diseases. BrainCare Oy (http://braincare.fi/) is a Tampere University of Technology spin-off company founded in 2013 to deliver personalized solutions to improve the quality of life of epilepsy patients. The organization recently concluded clinical trials for a novel mobile application and supporting solutions for long-term monitoring for epileptic patients. The ADNI was launched in 2003 as a public-private partnership, led by Principal Investigator Michael W. Weiner, MD. The primary goal of ADNI has been to test whether serial MRI, positron emission tomography (PET), other biological markers, and clinical and neuropsychological assessment can be combined to measure the progression of mild cognitive impairment and early Alzheimer’s disease.

### Test data

The test data consist of three hundred MRI slices extracted from 15 T2 and 10 T1 MRI volume data. In the results section we demonstrate the efficacy of our proposed method with six MRI volume data; three from NeuroRx, two from BrainCare and one from ADNI. The dataset from NeuroRx are one T2 weighted and two conventional T1 weighted. Each volume data contain 60 slices with dimension 256×256 pixels and 2.4 mm thickness. The T2 weighted and one of the T1 weighted MRI data are without any perceived distortion. The slices in the other T1 weighted data were originally acquired with various configurations of bias fields. There are two 50-slice, T2 weighted data without perceived distortion from BrainCare Oy. Each slice has dimension 448×408 pixels and 2.6 mm thickness. The remaining data is T1 magnetization-prepared rapid gradient echo (MPRAGE) pulse sequence from ADNI. It contains 180 slices. Each slice has dimension 190×160 pixels and 1.2 mm thickness.

Performance evaluation was carried out in six categories. The first three categories evaluate the data in their original form of acquisition. They are T2 volume data without perceived distortion, T1 volume data without perceived distortion and T1 volume data degraded by bias fields. In the remaining three categories, three different types of degradation; circular blur, motion blur and Rician noise at different levels were artificially induced on the test data.

An image was degraded with circular blur by convolution with space-varying pillbox function. The parameter of circular blur is determined from the radial distance, in pixels, of the pillbox function. Motion blur degradation was done by a filter which approximates the linear motion of a camera. Motion blur is defined by linear and angular distances in pixels and degrees, respectively. Rician noise level was computed from the magnitude of Gaussian noise added to the real and imaginary components of the image. The real and imaginary components of the image are generated by making two duplicte copies of the image. Rician noise parameter is defined by the percentage of the maximum pixel intensity level in the image [56]. The different levels of circular blur, motion blur and Rician noise degradation were defined by scaling the respective parameters from 1 to 15.

### Subjective Evaluation

In order to determine how our proposed method correlate with the human visual system we conducted subjective experiments with human observers. The experiment was aided by **QuickEval** [57], a web-based tool for psychometric image evaluation provided by the Norwegian Colour and Visual Computing Laboratory (www.colourlab.no/quickeval) at the Norwegian University of Science and Technology, Gjøvik, Norway. The observers are one radiologist and one MRI reader. MRI reader is a trained professional with experience working on MRI images that are affected by pathology [33]. Mean opinion score (MOS) subjective experiment was chosen for the validation study because it is simple and popular. Mean opinion score is the average of the quality scores assigned to an image by multiple viewers [58]. The six categories of the objective experiment was used for the subjective experiment. The observer assigns a score between 0 and 100, in steps of 1 to each slice. In the category of MRI volume data with artificially induced distortion, each observer was first presented with an undistorted version of an MRI slice, followed by increasing distortion levels of the original slice. The distorted levels are 5, 10 and 15. The relationship between our objective results and the score assigned by human observers was determined using the spearman’s rank correlation coefficient *ρ* [59]:

1 $$ \rho=1 - \frac{6\sum d^{2}}{n^{3} - n}  $$

where *n*, the number of observations is the total number of slices contained in all the volume data in each category of the experiment, *d* is the difference between the two ranks of each observation.

### Notational definitions

Here we explain and define the four binary images derived from the grayscale and contrast feature images. Each binary version is identified by a four-word name. The first three words describe the image moment of the feature image used as the global threshold to derive the binary image. The last word describe the original feature image before it was transformed to the binary domain. Each binary version is denoted by $J_{I}^{M}$. The superscript denote the image moment *M* that was set as the global threshold to derive the binary image. The subscript is the original feature image *I* before transformation to the binary domain. Table 1 is a summary of the notational definitions.

**Table 1:** Notational definitions for the four binary feature images used for quality evaluation of brain MRI images

BINARY FEATURE IMAGE	FEATURE ACRONYM	FEATURE NOTATION	THRESHOLD
First Grayscale Moment Grayscale	FGMG	$J_{I_{d}}^{\mu _{d}}$	(*I*_*d*_>*μ*_*d*_)
First Grayscale Moment Contrast	FGMC	$J_{I_{c}}^{\mu _{d}}$	(*I*_*c*_>*μ*_*d*_)
First Contrast Moment Contrast	FCMC	$J_{I_{c}}^{\mu _{c}}$	(*I*_*c*_>*μ*_*c*_)
First Contrast Moment Grayscale	FCMG	$J_{I_{d}}^{\mu _{c}}$	(*I*_*d*_>*μ*_*c*_)

#### First grayscale moment grayscale binary image

First Grayscale Moment Grayscale (**FGMG**) binary image $J_{I_{d}}^{\mu _{d}}$ is defined as the threshold version of the grayscale feature image *I*_*d*_ at global threshold *μ*_*d*_:

2 $$ J_{I_{d}}^{\mu_{d}}= \left\{\begin{array}{ll} 1 & \textbf{if} \quad I_{d} > \mu_{d}\\ 0 & \textbf{otherwise} \end{array}\right.  $$

where *μ*_*d*_ is the first moment of the grayscale feature image. This image can be regarded as the brightness quality descriptor for the observed image because the number of bright pixels determines the luminosity of the image.

#### First contrast moment grayscale binary image

First Contrast Moment Grayscale (**FCMG**) binary image $J^{\mu _{c}}_{I_{c}}$ is defined as the binary version of the grayscale feature image *I*_*d*_ at global threshold *μ*_*c*_:

3 $$ J^{\mu_{c}}_{I_{d}}= \left\{\begin{array}{ll} 1 & \textbf{if} \quad I_{d} > \mu_{c}\\ 0 & \textbf{otherwise} \end{array}\right.  $$

The bright pixels measures the influence of the brightness quality attribute on the contrast quality attribute. It determines the number of grayscale pixels that contribute to the contrast quality attribute

#### First contrast moment contrast binary image

First Contrast Moment Contrast (**FCMC**) binary image $J^{\mu _{c}}_{I_{c}}$ is defined as the threshold version of the local contrast feature image *I*_*c*_ at global threshold *μ*_*c*_ :

4 $$ J^{\mu_{c}}_{I_{c}}= \left\{\begin{array}{ll} 1 & \textbf{if} \quad I_{c} > \mu_{c}\\ 0 & \textbf{otherwise} \end{array}\right.  $$

where *μ*_*c*_ is the first moment of the contrast feature image. This image can be regarded as the texture quality descriptor for the observed image.

#### First grayscale moment contrast binary image

First Grayscale Moment Contrast (**FGMC**) binary Image $J^{\mu _{d}}_{I_{c}}$ is defined as the binary version of the contrast feature image *I*_*c*_ at global threshold *μ*_*d*_:

5 $$ J^{\mu_{d}}_{I_{c}}= \left\{\begin{array}{ll} 1 & \textbf{if} \quad I_{c} > \mu_{d}\\ 0 & \textbf{otherwise} \end{array}\right.  $$

The number of bright pixels in this image describe the interaction between the brightness and contrast quality attributes in the observed image.

### Problem formulation

Classical quality attributes are generally adopted for simple images. The report in [60] recognize the need for better description of quality attributes in complex images. Contributions in the literature such as [61, 62] adopt terms such as luminance contrast, texture and texture contrast to describe quality attributes in specific complex images. Texture features has been widely applied to distinguish normal and abnormal structures in MRI images [63–65].

We regard MRI image as a two-tissue class complex image. With reference to a T2 weighted MRI slice the bright pixels describe the high density of edges that characterize the cortical gray matter and the boundaries between the different anatomical structures. The white matter and other anatomical structures other than the cortical gray matter are described by the dark pixels.

We assume that all the possible distortions in an image can be condensed into either space-invariant point spread function or multiplicative spatially varying factor $\mathcal {H}$ and random noise *n* according to the mathematical model of a 2D image acquisition process [66, 67] expressed by:

6 $$ I_{d}=\mathcal{H}I_{f} + n  $$

where *I*_*d*_ is the observed grayscale image and *I*_*f*_ is the underlying ideal image. In the absence of distortion and following the two-tissue class model, the observed image and its local contrast feature image are replica of the underlying ideal image:

7 $$ I_{d}=I_{c}=I_{f}  $$

Based on Eq. 7 the local contrast feature image and the observed image will have same pixel intensity level:

8 $$ \mu_{d}=\mu_{c}  $$

We hereby propose four quality scores. They are luminance contrast, texture, texture contrast and lightness. Each quality score is derived from the structural matching of relevant pair of binary feature images. The structural matching are described as pixel-wise structural matching and as edge-pixel structural matching. Pixel-wise and edge-pixel structural matching compares all the corresponding pixels and only edge pixels, respectively in the foreground of both images.

#### Luminance contrast quality score

The structural matching between edge pixels in **FGMG** and corresponding edge pixels in **FCMG** expressed as:

9 $$ q_{11}=J_{I_{d}}^{\mu_{d}} \cap J_{I_{d}}^{\mu_{c}}  $$

measures how well the brightness quality attribute can be used to gauge the contrast quality attribute. This gives the luminance contrast quality score:

10 $$ q1= \frac{n_{q_{11}}}{\max \left(n_{J_{I_{d}}^{\mu_{d}}},n_{J_{I_{d}}^{\mu_{c}}}\right)}  $$

where $n_{q_{11}}$ is the number of bright pixels common to both **FGMG** and **FCMG** and the denominator is the highest number computed from the number $n_{J_{I_{d}}^{\mu _{d}}}, n_{J_{I_{d}}^{\mu _{c}}}$ of bright pixels in both feature images. The number of bright pixels in both images are compared and used as denominator to ensure that the quality index *q*_1_≤1.

#### Texture quality score

The structural matching between edge pixels in **FGMC** and corresponding edge pixels in **FCMC** expressed as

11 $$ q_{22}=J_{I_{c}}^{\mu_{d}}\cap J_{I_{c}}^{\mu_{c}}  $$

measures how well the brightness quality attribute can be used to gauge the texture quality attribute. This gives the texture quality score:

12 $$ q2= \frac{n_{q_{22}}}{\max \left(n_{J_{I_{c}}^{\mu_{d}}},n_{J_{I_{c}}^{\mu_{c}}}\right)}  $$

where $n_{q_{22}}$ is the number of bright pixels common to both **FGMC** and **FCMC** and the denominator is the highest number computed from the number $n_{J_{I_{c}}^{\mu _{d}}}, n_{J_{I_{c}}^{\mu _{c}}}$ of bright pixels in both feature images.

#### Texture contrast quality score

The pixel-wise structural similarity matching between **FGMC** and **FCMC** expressed as

13 $$ q_{33}=J_{I_{c}}^{\mu_{d}}\cap J_{I_{c}}^{\mu_{c}}  $$

gives the texture contrast quality score expressed as:

14 $$ q3= \frac{n_{q_{33}}}{n_{t}}  $$

where $n_{q_{33}}$ is the number of dark and bright pixels common to both **FGMC** and **FCMC**, and *n*_*t*_ is the number of foreground pixels. In an ideal image where quality distortions are absent, Eq. 8 holds. Therefore the texture contrast quality score expressed by Eq. 14 is 1. For real images, texture contrast quality score is determined by the disparity between *μ*_*d*_ and *μ*_*c*_.

#### Lightness quality score

The pixel-wise structural similarity matching between **FGMG** and **FCMG** expressed as

15 $$ q_{44}=J_{I_{d}}^{\mu_{d}} \cap J_{I_{d}}^{\mu_{c}}  $$

gives the lightness quality score expressed as:

16 $$ q4 = \frac{n_{q_{44}}}{n_{t}}  $$

where $n_{q_{44}}$ is the number of dark and bright pixels common to both **FGMG** and **FCMG**. The disparity between *μ*_*d*_ and *μ*_*c*_ determines the lightness quality score.

#### Total quality score

The total quality score *Q* is the weighted sum of the four quality scores:

17 $$ Q= w_{q_{1}}q_{1} + w_{q_{2}}q_{2} + w_{q_{3}}q_{3} + w_{q_{4}}q_{4}  $$

where $w_{q_{1}}, w_{q_{2}}, w_{q_{3}}, w_{q_{4}}$ are the perceptual weights assigned to luminance contrast, texture, texture contrast and lightness quality attributes, respectively.

### Implementation

**Fig. 1:**
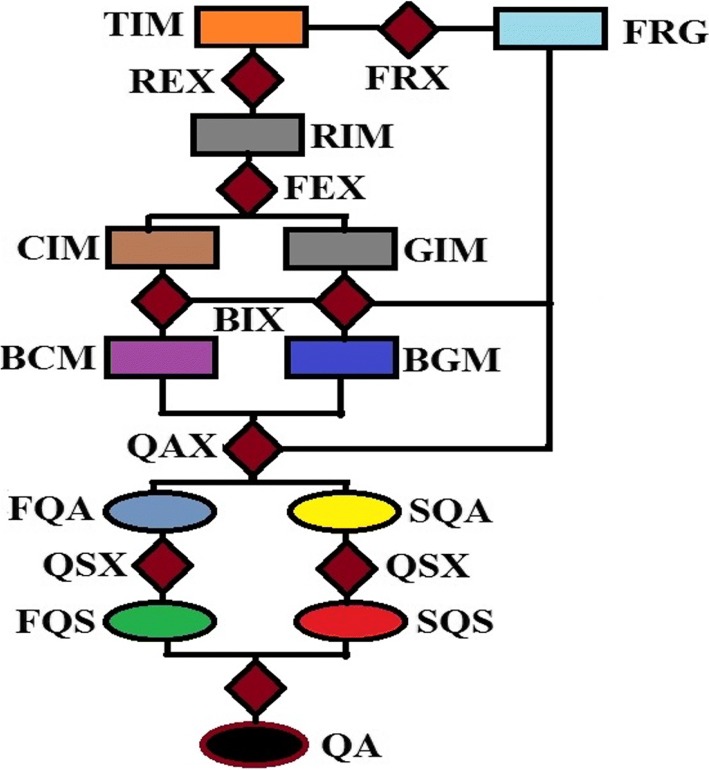
The flow chart for post-acquisition quality evaluation of a brain MRI slice. Foreground **FRG** is extracted **FRX** from the test image **TIM**. The test image is rescaled **REX** so that its pixel intensity levels is between 0 and 1. Two feature images, local contrast feature image **CIM** and grayscale image **GIM** are extracted from the rescaled image **RIM**. Global thresholding transforms the feature images into four binary feature images (only two **BCM** and **BGM** of the four binary feature images are shown). Determination of quality attributes **QAX** gives luminance contrast, texture contrast, texture and lightness quality attributes (only two, **FQA** and **SQA** of the four quality attributes are shown). The quality attributes are determined by matching relevant combinations of the binary feature images. Computation of quality score **QSX** for each quality attribute gives luminance contrast, texture, texture contrast and lightness quality scores (only two, **FQS** and **SQS**, of the four quality scores are shown). The total quality score **QA** is the weighted sum of the scores assigned to each quality attribute

**Fig. 2:**
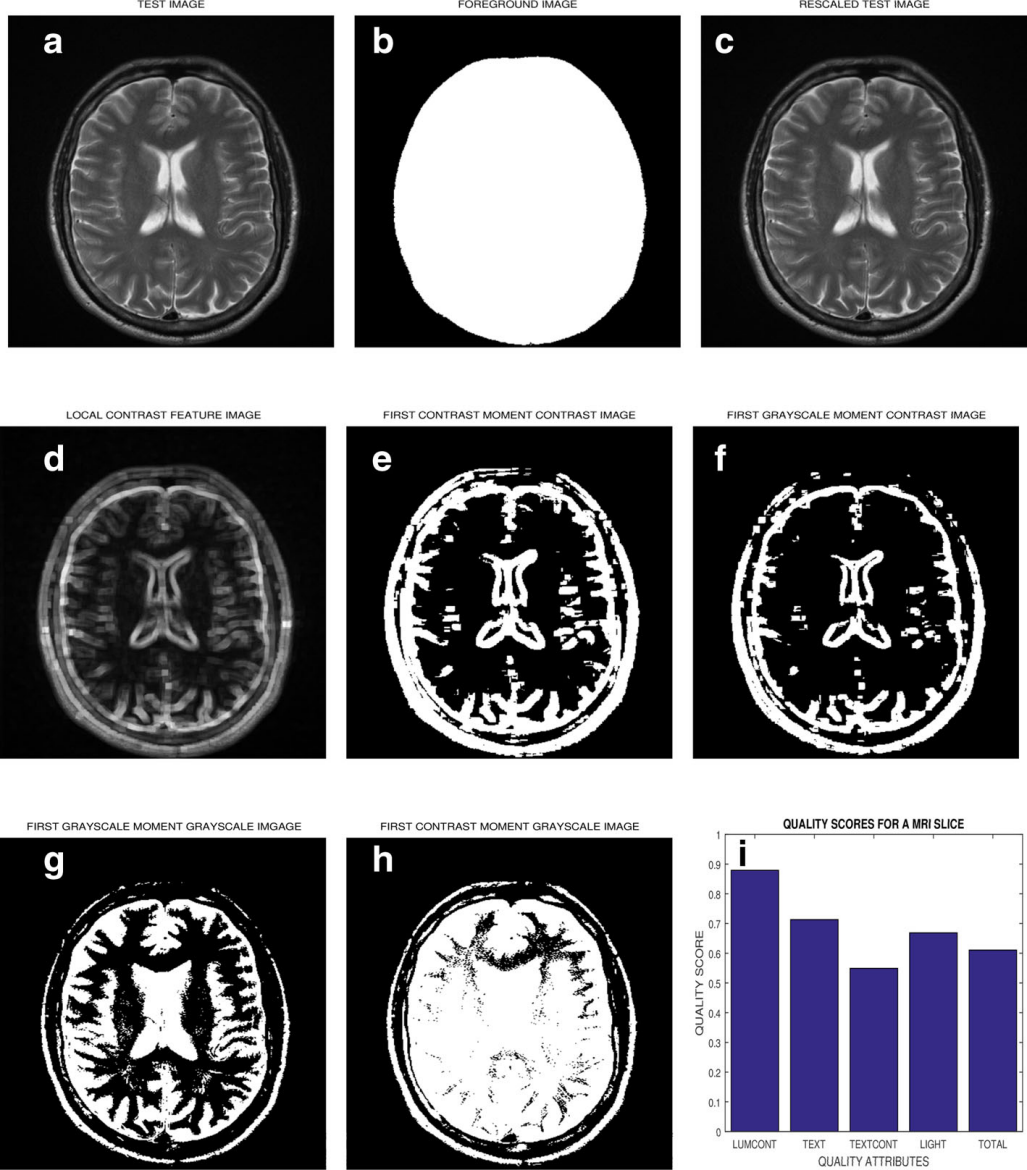
The different stages of the algorithm for post-acquisition quality evaluation of a brain MRI slice. **a** The test image has its (**b**) foreground extracted. **c** The test image in (**a**) has its pixel intensity levels rescaled to lie between 0 and 1. **d** Grayscale and contrast feature images are extracted from the test image. Duplicating the rescaled image in (**c**) extracts the grayscale image at no computational cost. **e**, **f**, **g** and **h** are the four binary feature images generated by using the first moments of the feature images in (**c**) and (**d**) as global thresholds. **i** Luminance contrast, texture, texture contrast, lightness and total quality scores are computed by matching relevant pairs of the feature images in (**e**), (**f**), (**g**) and (**h**)

The algorithm was implemented in the MatLab computing environment. The flow chart of Fig. 1 and the images displayed in Fig. 2 describe the six steps to implement the algorithm. The first two steps, foreground extraction and pixel intensity rescaling are meant to normalize data from the three different sources.

#### Step 1 - Foreground extraction

The first task in the implementation of the algorithm is to extract **FRX** the foreground **FRG** shown in Fig. 2b. The foreground pixels describe the actual anatomical structures of the test image **TIM** shown in Fig. 2a. Foreground extraction is a very critical step because the number of foreground pixels are required as input at step 4 where the binary feature images are generated and at step 6 for the computation of the quality scores.

#### Step 2 - Intensity rescaling

The intensity level of the test image **TIM** is rescaled **REX** to lie between 0 and 1 so that the rescaled test image **RIM** in Fig. 2c can be regarded as a blurred version of an ideal binary image according to the novel work in [68].

#### Step 3 - Feature image extraction

The rescaled test image **RIM** is convolved with a local range filter to extract **FEX** local contrast feature image **CIM** shown in Fig. 2d. The algorithm is sensitive to the size of filter. Larger filter size causes loss of fine details while smaller filter size will result in loss of spatial coherence in the filtered image [69]. For the aforementioned reasons and based on our experience during performance evaluation of the proposed algorithm we recommended filter width of 3×3 pixels, 5×5 pixels and 7×7 pixels for images with dimension comparable to 250×250 pixels, 350×350 pixels and 450×450 pixels, respectively. The mean of the local contrast image is computed with reference to the foreground pixels. The gray level feature image **GIM** is extracted at no computational cost by making a duplicate copy of the rescaled test image shown in Fig. 2c.

#### Step 4 - Binary feature images

According to Eqs. 2 - 5 four binary feature images (**FGMG**, **FCMG**, **FCMC** and **FGMC**) shown in Fig. 2e, f, g and h, respectively are generated from the two feature images; **CIM** and **GIM**. In the flow chart the four binary feature images are represented with two symbols; **BCM** and **BGM**.

#### Step 5 - Quality attribute

Quality attributes **QAX** of the test image are determined from similarity matching of relevant pairs of binary feature images generated in step 4 according to Eqs. 9, 11, 13 and 15. In the flow chart two symbols **FQA**, **SQA** are used to represent the four quality attributes.

#### Step 6 - Quality score

Quality score **QSX** for each quality attribute displayed in Fig. 2i is computed according to Eqs. 10, 12, 14 and 16. Two symbols **FQS**, **SQS** in the flow chart represent the quality scores. The total quality score **QA** is the weighted sum of each quality attribute. The perceptual weight assigned to each quality attribute is arbitrary but is based on [70] which reports that texture contrast quality attribute contributes approximately 10 times more to the generation of saliency in natural scenes than luminance contrast. Throughout the study the weight assigned to each quality attribute was fixed as follows; $w_{q_{1}}=0.1$, $w_{q_{2}}=0.1$, $w_{q_{3}}=0.7$ and $w_{q_{4}}=0.1$.

## Results

The images and the tables in this section demonstrate some results in the six different categories of the objective and subjective performance evaluation of our proposed quality evaluation method.

### Good Quality T2 MRI Volume Data

**Fig. 3:**
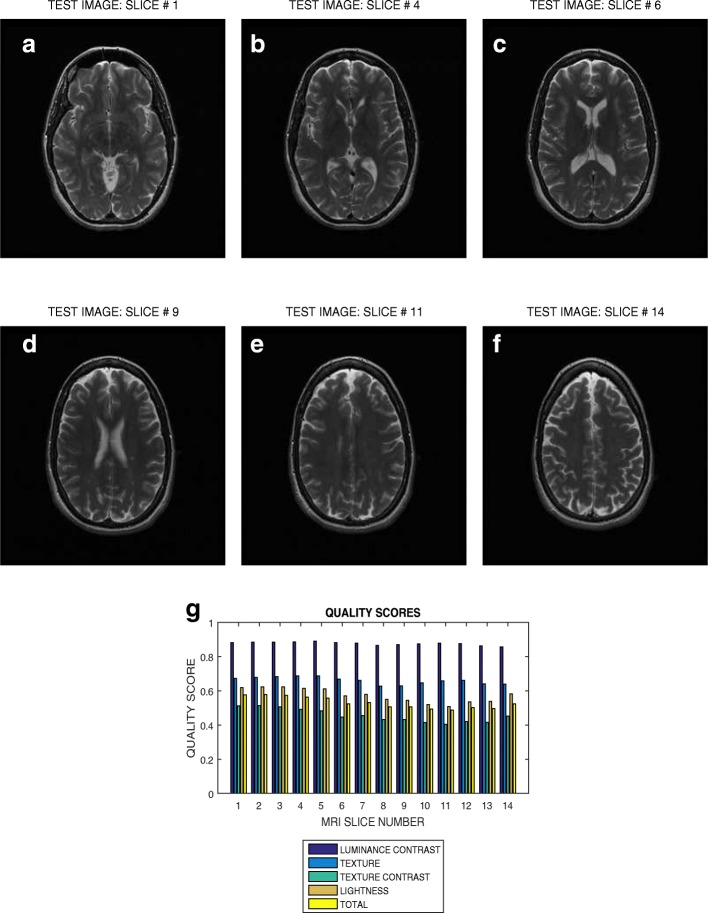
Six slices with indices (**a**) 1, (**b**) 4, (**c**) 6, (**d**) 9, (**e**) 11 and (**f**) 14 in a T2 weighted MRI volume data from NeuroRx Research Inc, (**g**) luminance contrast, texture, texture contrast, lightness and total quality scores of 14 successive slices in the MRI volume data

**Fig. 4:**
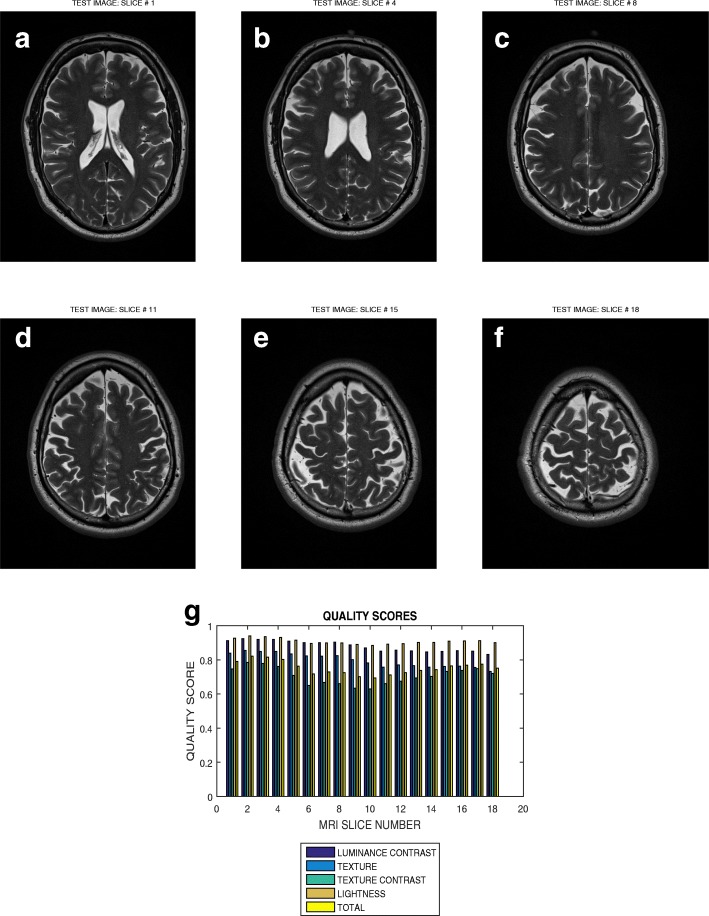
Six slices with indices (**a**) 1, (**b**) 4, (**c**) 8, (**d**) 11, (**e**) 15 and (**f**) 18 in a T2 weighted MRI volume data from BrainCare Oy, (**g**) luminance contrast, texture, texture contrast, lightness and total quality scores of 18 successive slices in the MRI volume data

Six slices in T2 weighted MRI volume data from NeuroRx and BrainCare are shown in Figs. 3a – f and 4a – f, respectively. The plots in Figs. 3g and 4g are the luminous contrast, texture, texture contrast, lightness and total quality scores for 14 and 18 successive slices in the MRI volume data.

### Good Quality T1 MRI Volume Data

**Fig. 5:**
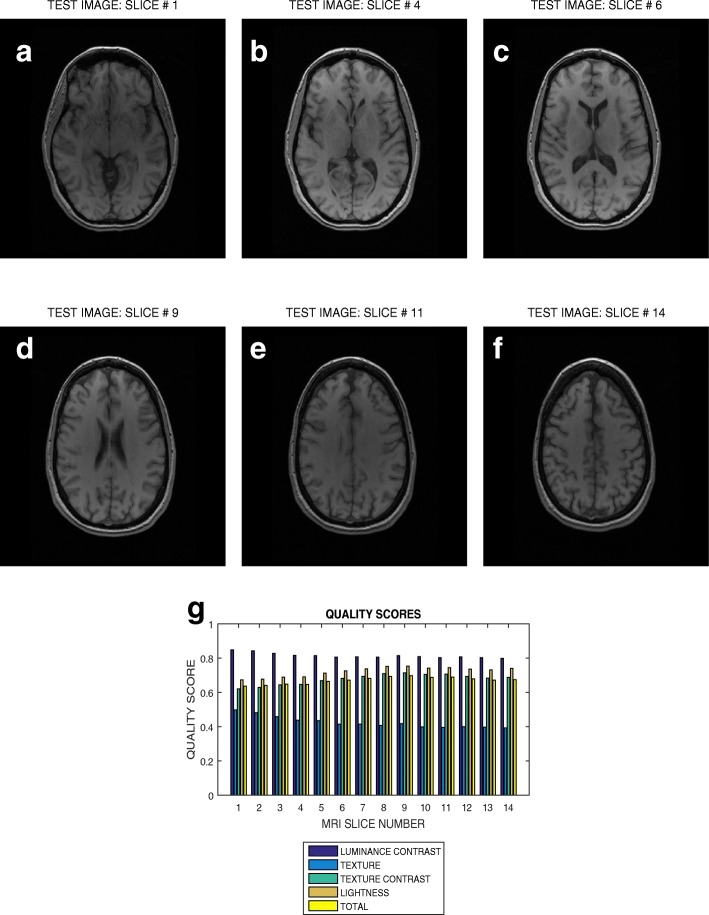
Six slices with indices (**a**) 1, (**b**) 4, (**c**) 6, (**d**) 9, (**e**) 11 and (**f**) 14 in a T1 weighted MRI volume data from NeuroRx Research Inc., (**g**) luminance contrast, texture, texture contrast, lightness and total quality scores of 14 successive slices in the MRI volume data

**Fig. 6:**
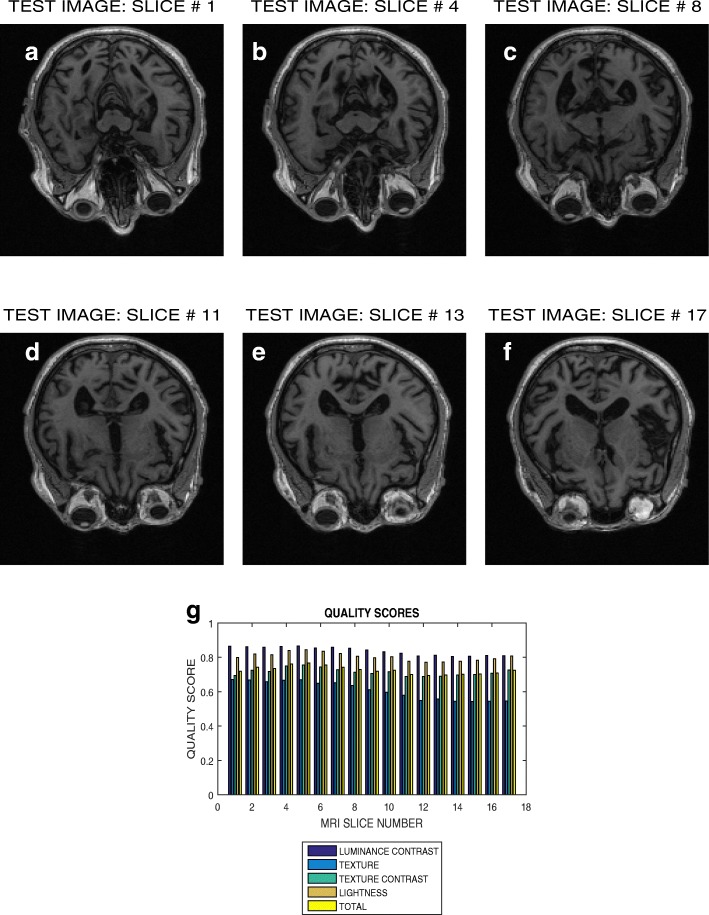
Six slices with indices (**a**) 1, (**b**) 4, (**c**) 8, (**d**) 11, (**e**) 13 and (**f**) 17 in a T1 MPRAGE pulse sequence MRI volume data from ADNI, (**g**) luminance contrast, texture, texture contrast, lightness and total quality scores of 17 successive slices in the MRI volume data

The images in Figs. 5a – f and 6a – f are slices in T1 weighted MRI volume data from NeuroRx and ADNI, respectively. Their respective luminous contrast, texture, texture contrast, lightness and total quality scores are shown in Figs. 5h and 6h, respectively.

### Circular Blur

**Fig. 7:**
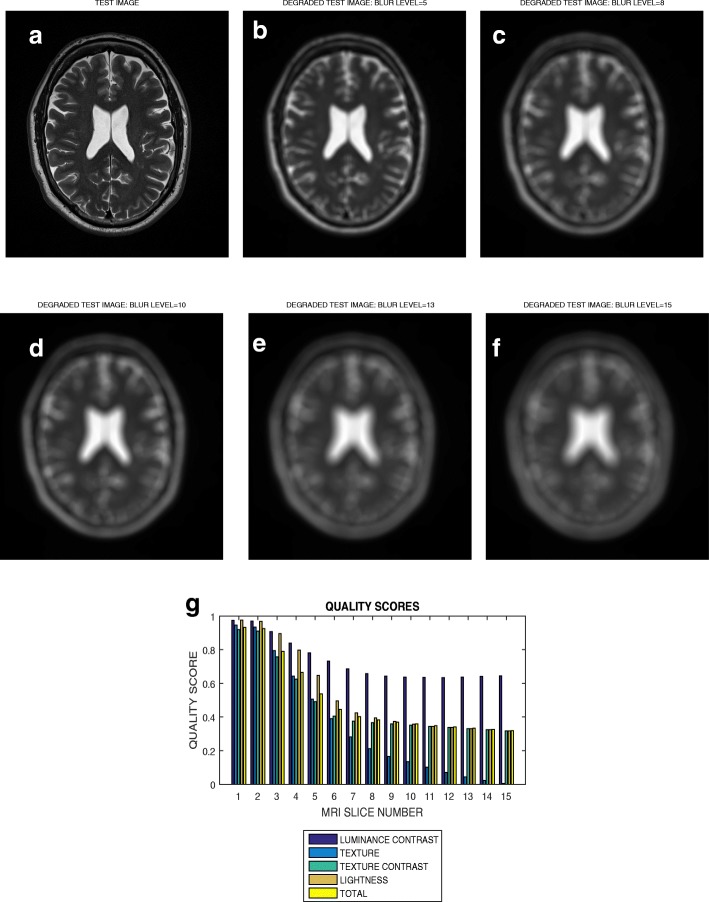
**a** A T2 weighted slice degraded by circular averaging filter of radius (**b**) 5, (**c**) 8, (**d**) 10, (**e**) 13 and (**f**) 15 pixels. **g** variation of the luminance contrast, texture, texture contrast, lightness and total quality scores with blur levels increasing from 1 to 15

The image in Fig. 7a is a slice from a T2 weighted MRI volume data from BrainCare. The images shown in Fig. 7b – f are the same image in Fig. 7a but were blurred with circular averaging filter of radius 5 pixels, 8 pixels, 10 pixels, 13 pixels and 15 pixels, respectively. The luminous contrast, texture, texture contrast, lightness and total quality scores for blur levels from 1 pixels to 15 pixels are shown in Fig. 7g.

### Motion blur

**Fig. 8:**
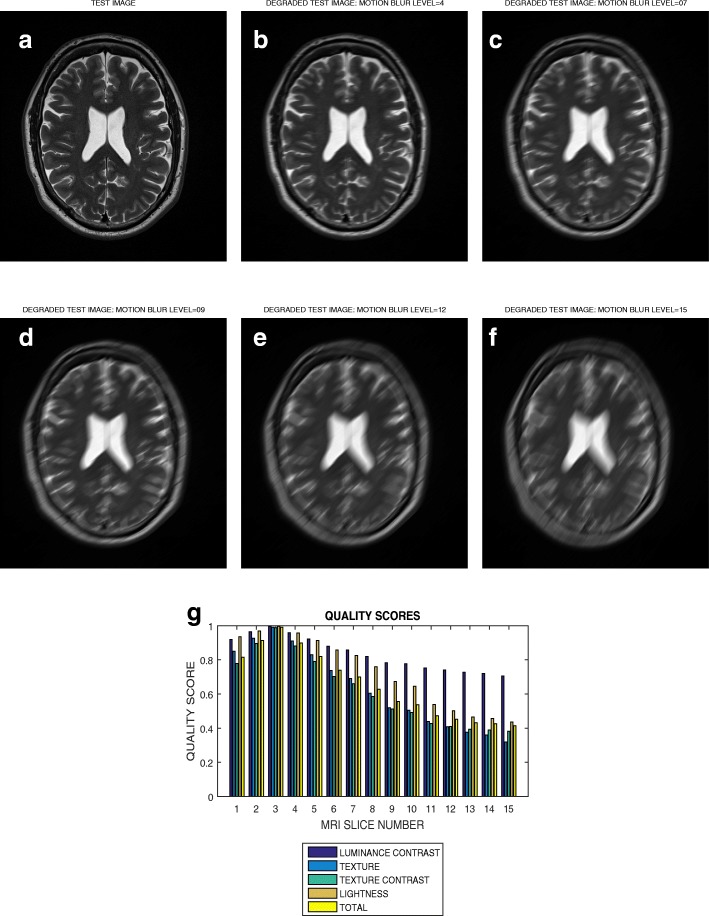
**a** A T2 weighted slice and its degraded versions at motion blur levels (**b**) 5, (**c**) 8, (**d**) 10, (**e**) 13 and (**f**) 15 pixels, (**g**) variation of the luminance contrast, texture, texture contrast, lightness and total quality scores with blur levels increasing from 1 to 15

A slice in a T2 weighted MRI volume data from BrainCare is shown in Fig. 8a. Its motion blurred versions are shown in Fig. 8b – f for motion blur levels of 4 pixels, 7 pixels, 9 pixels, 12 pixels and 15 pixels, respectively. The plot of the motion blur levels from 1 pixels to 15 pixels versus luminous contrast, texture, texture contrast, lightness and total quality scores are displayed in Fig. 8g.

### Noise

**Fig. 9:**
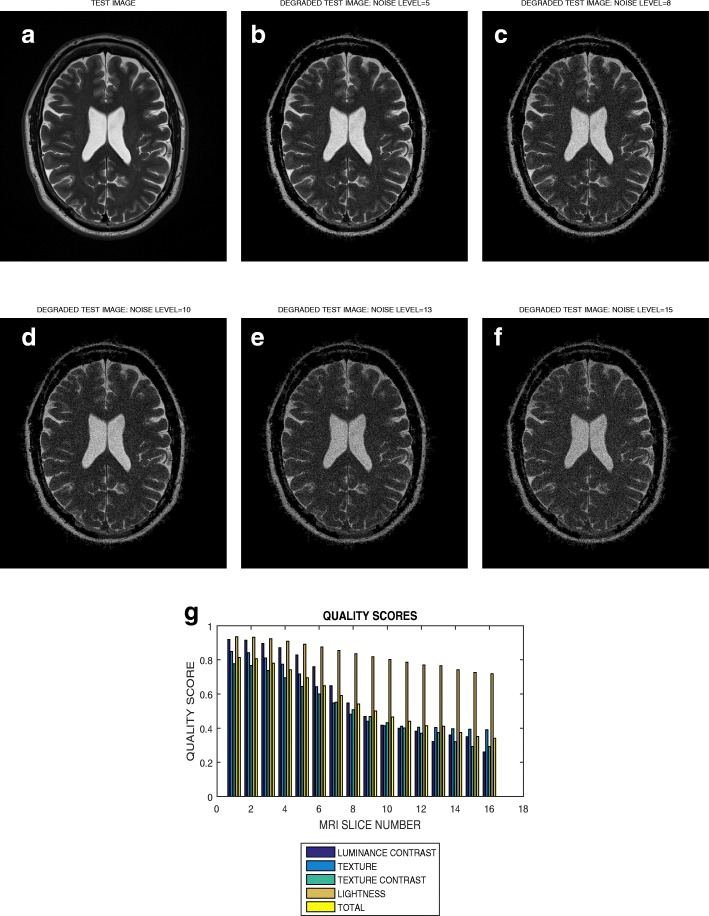
**a** A T2 weighted slice and its degraded versions at Rician noise levels (**b**) 5, (**c**) 8, (**d**) 10, (**e**) 13 and (**f**) 15 percent, (**g**) variation of the luminance contrast, texture, texture contrast, lightness and total quality scores with noise levels increasing from 1 to 15

The image in Fig. 9a is a slice in the T2 weighted volume data from BrainCare. Its Rician noise degraded versions are shown in Fig. 9b – f for noise levels of 5, 8, 10, 13 and 15%, respectively. The plot of luminous contrast, texture, texture contrast, lightness and total quality scores for noise levels from 1 to 15 are displayed in Fig. 9g.

### Bias Fields

**Fig. 10:**
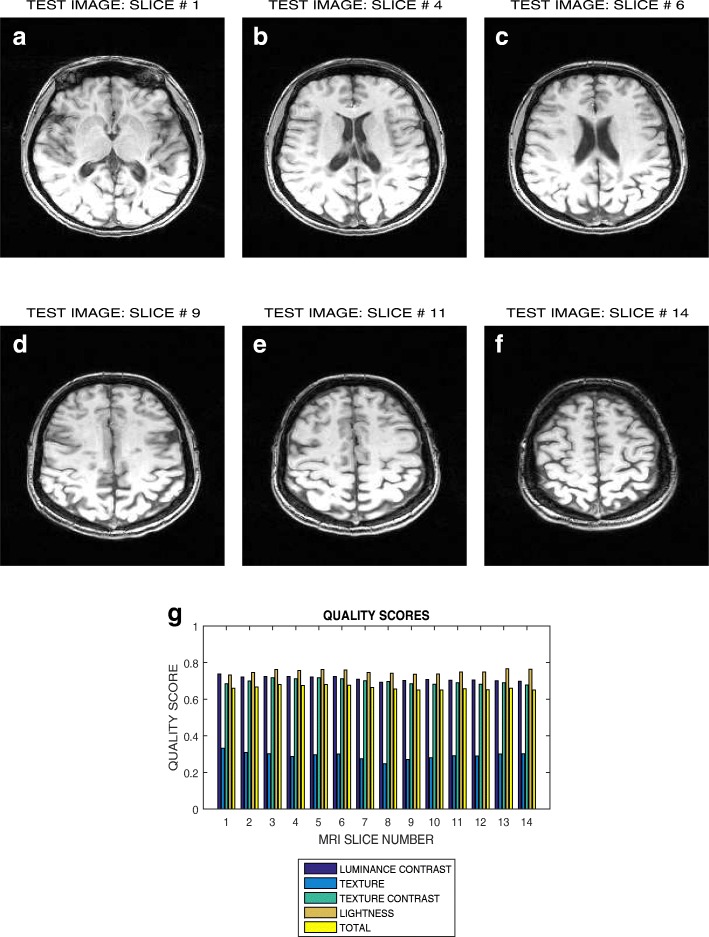
Six slices with indices (**a**) 1, (**b**) 4, (**c**) 6, (**d**) 9, (**e**) 11, (**f**) 14 in a T1 weighted MRI volume data degraded by different configurations of bias fields, (**g**) luminance contrast, texture, texture contrast, lightness and total quality scores for each slice in the volume data

Six images shown in Fig. 10a – f are slices in a T1 weighted MRI volume data from NeuroRx. They were originally degraded with different configurations of bias fields during acquisition stage. The luminous contrast, texture, texture contrast, lightness and total quality scores for 14 successive slices in the volume data is shown in Fig. 10g.

### Validation of Results

Results from the subjective evaluation of our proposed method are tabulated in Tables 2, 3, 4 and 5. In Table 2 are the results for T2 and T1 MRI volume data without perceived distortion as well as T1 volume data that were originally acquired with bias fields. Tables 3, 4 and 5 are the results for blurring with circular averaging filter, blurring with motion blur and degradation with Rician noise, respectively.

**Table 2:** Validation results for T2 and T1 MRI images without perceived distortion and T1 MRI images degraded by bias fields

MRI volume data	Number of slice	Average objective score	Average subjective score	Objective- subjective correlation coefficient	Inter-observer correlation coefficient
T2 without perceived distortion	50	0.80	0.70	0.84	0.90
T1 without perceived distortion	50	0.70	0.60	0.80	0.85
T1 degraded by bias fields	50	0.40	0.30	0.75	0.75

**Table 3:** Validation results for T2 MRI images degraded by blurring with circular averaging filter

Distortion level	Number of slice	Average objective score	Average subjective score	Objective- subjective correlation coefficient	Inter-observer correlation coefficient
0	50	0.80	0.70	0.84	0.85
5	50	0.70	0.70	0.72	0.80
10	50	0.50	0.40	0.75	0.75
15	50	0.30	0.30	0.70	0.65

**Table 4:** Validation results for T2 MRI images degraded by blurring with circular motion blur

Distortion level	Number of slice	Average objective score	Average subjective score	Objective- subjective correlation coefficient	Inter-observer correlation coefficient
0	50	0.80	0.70	0.84	0.85
5	50	0.70	0.60	0.80	0.85
10	50	0.40	0.30	0.75	0.80
15	50	0.30	0.30	0.70	0.70

**Table 5:** Validation results for T2 MRI sequence images degraded by Rician noise

Distortion level	Number of slice	Average objective score	Average subjective score	Objective- subjective correlation coefficient	Inter-observer correlation coefficient
0	50	0.80	0.70	0.84	0.8
5	50	0.80	0.60	0.70	0.8
10	50	0.70	0.70	0.70	0.75
15	50	0.50	0.40	0.60	0.65

## Discussion

### Evaluation across good quality T2 MRI images

The plots in Figs. 3 and 4 show variation in the quality scores across slices in the T2 weighted MRI volume data from NeuroRx and BrainCare, respectively. The luminance contrast, texture. texture contrast and lightness quality scores for the images from NeuroRx vary from 0.85 to 0.90, 0.65 to 0.70, 0.40 to 0.50 and 0.50 to 0.60, respectively. Corresponding quality scores for the images from BrainCare are 0.90 to 0.95, 0.80 to 0.85, 0.65 to 0.75 and 0.90 to 0.95. The results show that our proposed method objectively evaluate the variations in image quality in the slices contained in perceived good quality T2 weighted MRI volume data.

### Evaluation across good quality T1 MRI images

The plot in Fig. 5 show that the average luminance contrast, texture. texture contrast, lightness and total quality scores of the slices in the NeuroRx T1 MRI volume data are 0.80, 0.45, 0.65, 0.70 and 0.50, respectively. Corresponding quality scores for the same type of MRI volume data from ADNI are 0.82, 0.65, 0.70, 0.80 and 0.70, respectively. Since both data were tagged as good quality images before the experiment, it can be said that our proposed method demonstrated good objective evaluation across slices in perceived good quality T1 weighted MRI data.

### Evaluation across poor quality T1 MRI images

The average luminance contrast, texture. texture contrast, lightness and total quality scores for the images degraded by different configurations of bias fields are 0.75, 0.30, 0.7, 0.75 and 0.42, respectively (See Fig. 10). A cursory visualization of the six slices in Fig. 10 shows that there is contrast between the different anatomical structures but there is evidently loss of edges which is a measure of details that describe each anatomical structure within the image. The texture quality score of 0.30 recorded by our proposed method can be said to be a very good objective evaluation of the presence of bias field in the image. The presence of bias field can cause an automated image analysis system to erroneously misclassify the structures in the image. The texture quality index of 0.3 recorded by our proposed method can draw the attention of image analysis experts to carry out restoration on the degraded images before further processing.

### Evaluation across different levels of distortion

The plot in Fig. 7 shows that the total quality score successively decrease from 0.95 to 0.15 for circular blur levels that vary from 1 to 15 in a T2 weighted slice. For the same slice in Fig. 8 there is an initial increase of total quality score for motion blur that increase from 1 to 3. This initial increase in quality score can be attributed to extraneous features which mimic image details during the initial introduction of motion blur. Thereafter there is successive decrease in quality score from 0.98 to 0.40 for blur levels that vary from 3 to 15. The slice in Fig. 9a is the same slice shown in Figs. 7a and 8a but degraded by Rician noise level that vary from 1 percent to 15 percent. The plot in Fig. 9g show successive decrease in total quality score from 0.85 to 0.42. These results show that our proposed method demonstrate promising performance in the evaluation of images with different perceptual quality.

### Evaluation across different quality attributes

The slice in Fig. 9a is one of the slices in the T2 weighted volume data shown in Fig. 4. The plot in Fig. 4g indicates that, in the absence of distortion, the average total quality scores vary from from 0.8 to 0.85. In the presence of degradation by circular motion blur, motion blur and noise the average total quality scores varies from 0.95 to 0.15, 0.98 to 0.42 and 0.85 to 0.42. This is an indication that our proposed method can objectively evaluate different quality attributes for different levels of distortion in an image.

### Correlation with subjective evaluation by human observers

The validation results in Tables 2, 3, 4 and 5 show that our proposed objective method has good correlation with human visual perception. Correlation between the scores assigned by the observers varies with the image sequence and the level of distortion in the images. The validation results shows that there is a trend of better agreement between the human observers at lower levels of distortion than at higher levels of distortion. An example is T2 MRI images degraded by blurring with circular averaging filter shown in Table 3. In the absence of distortion, the inter-observer correlation coefficient is 0.85. The corresponding inter-observer coefficients for 5, 10 and 15 distortion levels are 0.8, 0.75 and 0.65, respectively. In Table 2 the correlation coefficient of *ρ*≥0.80 for MRI volume data without perceived distortion show that there is very good correlation between quality scores recorded by our proposed method and the quality scores assigned by human observers. There is high correlation of 0.75 for validation result of T1 weighted volume data that were originally acquired with bias fields. The validation results table show that for different levels of circular blur, motion blur and Rician noise, the inter-observer correlation coefficient *ρ*≥0.65, and the objective-subjective correlation coefficient is *ρ*≥0.60.

### Standardization of quality metric

There is a clearly defined lower and upper limit of quality index. The lower limit is 0 for an extremely degraded image and upper limit of 1 for an ideal image. Quality index for a real MRI image lies between 0 and 1. This quality evaluation index is applied across images derived from different clinical trial sites, different scanners and different acquisition protocols. Thus a remarkable characteristic of our proposed method is the standardization of quality metric.

### Cut-off quality index

Our proposed quality metric system predict different quality scores for different MRI sequences. There are three reasons to suggest that the cut-off quality metrics to determine images of acceptable quality should be flexibly applied across different MRI sequences. First, different MRI sequences such as T2 and T1 that are without perceived distortion reveal different levels of structural information. Second, different MRI sequence images are acquired for different tasks. This leads to the philosophy of task-based quality evaluation [71, 72]. Third, it is more realistic to compare the quality of similar MRI sequence images acquired for the same task.

The quality measure is applied slice-wise across the MRI volume data. Generally, quality scores of slices contained in a MRI volume data lies within a narrow band of quality index. Based on our experience during the performance evaluation of our proposed algorithm and with specific importance to the opinion of human observers we recommend a cut-off quality index of 0.40 and 0.45 for T1 and T2 weighted MRI images, respectively. Although our methodology allows the computation of the total quality score for individual slices we hereby emphasize that the cut-off quality metric does not suggest rejection of individual slices. The recommended cut-off quality metric is the total quality score computed from the average quality scores of the slices contained in the MRI volume data.

### Absence of comparative performance evaluation

Three characteristics of existing methods makes it difficult to include comparative performance evaluation in this report. First, existing methods adopt different distortion models. Second, there are no clearly defined lower and upper limits of quality indices. Third, there are many definitions of the popular quality models such as SNR.

### Limitations of proposed method and future work

Three characteristics of our proposed method limit its efficacy in real-life scenarios. First, is the two-tissue class model we adopt for MRI images. It excludes the ventricular system. The model assumes that the brain consists of only white matter and cortical gray matter structures. The fixed perceptual weights assigned to the four quality attributes throughout the study is a theoretical approach that is yet to be validated. The third limitation is that our proposed method predict image quality based on the assumption that distortion process have same effect on the different structures of the brain.

Future work will adopt a new model which accounts for all the three major anatomical structures of the brain. Segmentation algorithm will be incorporated to delineate the boundaries of the three major anatomical structures so that quality score prediction will be assigned to region-of-interest. The perceptual weights assigned to each quality attributes will be refined based on the subjective scores from human observers. We will also explore the use of higher moments as a basis to describe image quality attributes.

## Conclusions

There is increasing clinical interest in the use of brain MRI images for the study of human anatomy, treatment and diagnosis of diseases as well as the clinical trials of drugs for the treatment of neurological diseases. Post-acquisition image quality evaluation is necessary to re-evaluate and standardize the quality of brain MRI images acquired from different clinical trial sites across the globe. No-reference objective image quality assessment is highly desired in environment where large volumes of MRI data are processed. We propose a new method to evaluate the quality of brain MRI images. The proposed method will be suitable for fully automated environments because processing of the quality metrics is on binary images. Experimental results demonstrates that our proposed method had good correlation with human visual judgement and gives fairly accurate quality evaluation within and across good quality images and different levels of degradation.

## Abbreviations

ADNI: Alzheimer’s disease neuroimaging initiative
ANOVA: Analysis of variance
BCM: Binary feature image extracted from the local contrast feature image
BGM: Binary feature image extracted from the grayscale feature image
CIM: Contrast feature image
CNR: Contrast-to-noise ratio
FEX: Feature extraction
FRG: Foreground extraction
FRX: Foreground extraction
FQA: Luminance contrast and lightness quality attributes
FQS: Luminance contrast and lightness quality attributes
GIM: Grayscale feature image
MPRAGE: Magnetization-prepared rapid gradient echo
MRI: Magnetic resonance imaging
QA: Total quality score
QAX: Quality attribute extraction
QSX: Quality score extraction
REX: Intensity rescaling operation
RIM: Intensity rescaled image
RMSE: Root means square error
SNR: Signal-to-noise ratio
SQA: Texture and texture contrast quality attributes
SQS: Texture and texture contrast quality attributes
TIM: Test image
T2: Transverse relaxation
T1: Longitudinal relaxation
